# The influence of factors related to public health campaigns on vaccination behavior among population of Wuxi region, China

**DOI:** 10.3389/fpubh.2024.1498296

**Published:** 2025-01-10

**Authors:** Yang Ye, Anselm Ting Su

**Affiliations:** ^1^Wuxi Hospital of Traditional Chinese Medicine, Wuxi, China; ^2^Faculty of Medicine and Health Sciences, Universiti Malaysia Sarawak, Sarawak, Malaysia

**Keywords:** public health campaigns, vaccination behavior, socioeconomic status, geographical disparities, health campaign quality, vaccination service accessibility

## Abstract

**Background:**

Public health campaigns are essential for promoting vaccination behavior, but factors such as socioeconomic status, geographical location, campaign quality, and service accessibility influence vaccine uptake. In the Wuxi region of China, disparities in vaccination behavior are seen between urban and rural populations and among different socioeconomic groups. This study aims to explore the factors related to public health campaigns that affect vaccination behavior in Wuxi, contributing to better public health strategies.

**Methods:**

A cross-sectional survey was conducted among 750 participants in Wuxi, focusing on their perceptions of socioeconomic status, geographical location, health campaign quality, and vaccination convenience. The questionnaire was developed based on a literature review and expert input using the Delphi method. Data were analyzed using descriptive statistics, reliability and validity tests, correlation analysis, and regression analysis, employing both SPSS and R software.

**Results:**

Socioeconomic status, geographic location, campaign quality, and accessibility all significantly influence vaccination behavior. Higher socioeconomic backgrounds, urban residency, better campaign quality, and greater accessibility to vaccination services are positively correlated with higher vaccination uptake. Regression analysis revealed that public health campaigns and accessibility are particularly influential in promoting vaccination behavior.

**Conclusion:**

To improve vaccination rates, targeted strategies focusing on low socioeconomic groups, rural areas, and improving campaign quality and service accessibility are necessary. Public health campaigns should be clear, culturally relevant, and utilize multiple communication channels. Future research should address misinformation, explore behavioral economics, and integrate emerging technologies like AI to optimize vaccination efforts.

## 1 Introduction

In recent years, with the continuous and in-depth implementation of China’s Expanded Program on Immunization (EPI), vaccination coverage has seen a significant increase, particularly in urban areas. By October 2024, the average vaccination rate for national immunization program vaccines among school-age children nationwide had reached 99.13%, highlighting China’s notable achievements in the field of public health. However, despite the overall high vaccination rate, there remains a noticeable gap between urban and rural areas, especially in rural regions where vaccination rates and vaccine accessibility are still relatively low. The disparity between urban and rural areas is one of the prominent challenges facing China’s vaccination efforts. Urban residents typically have more convenient access to vaccination services, with better-developed medical infrastructure, allowing them to receive vaccines more quickly and easily. In areas like Wuxi, urban residents not only have easier access to vaccines but also possess a higher level of health awareness, which enables them to actively participate in vaccination campaigns (1). However, in rural areas, residents face a series of obstacles to vaccination, including a lack of medical resources, inconvenient transportation, and insufficient vaccine promotion. Due to the limited medical facilities in rural areas, many residents have to travel long distances to reach vaccination sites, and the waiting times and inconveniences during the vaccination process further reduce their willingness to get vaccinated. Additionally, the channels for information dissemination in rural areas are limited, and vaccine promotion campaigns often fail to reach all areas, affecting rural residents’ awareness and participation in vaccination efforts. Higher-income groups in China show higher levels of participation in vaccination. This demographic generally has greater health awareness, enabling them to promptly access the latest vaccine information, and they often have more financial resources and time to complete vaccination procedures. Moreover, higher-income groups usually have higher education levels, which allows them to better understand the necessity and importance of vaccination. In contrast, the vaccination behavior of lower-income groups is restricted by various factors. On one hand, the economic burden may lead them to prioritize other living expenses over vaccination, resulting in delays or neglect of vaccination. On the other hand, lower-income groups have fewer channels to obtain relevant health information, and their awareness of vaccines is relatively low. Coupled with a possible lack of trust in the government and healthcare institutions, this further affects their vaccination decisions (2). In China, public health promotion plays an important role in encouraging vaccination behavior. However, how to increase vaccination rates, particularly among rural and low-income populations, remains a key issue in the field of public health. First, the uneven distribution of medical resources is a major challenge. While urban areas have relatively developed medical facilities, rural areas still suffer from weak medical infrastructure, with insufficient vaccination sites and a lack of healthcare personnel, which directly affects the vaccination rate in rural areas. Secondly, the lack of information dissemination also limits the effectiveness of vaccine promotion. Public health campaigns often fail to comprehensively reach remote corners of rural areas, leaving some residents with insufficient awareness of the importance of vaccination.

Globally, public health campaigns have played a crucial role in promoting vaccination (3–7). Successful campaigns often employ strong communication strategies and community engagement, as seen in the eradication of smallpox and the Global Polio Eradication Initiative (8, 9). However, challenges such as cultural resistance, misinformation, and logistical barriers can undermine efforts, as evidenced by the dengue vaccine controversy in the Philippines (10, 11). The role of digital media has also become prominent, both as a tool for spreading accurate information and a platform for misinformation, particularly during the COVID-19 pandemic (12). Although recent campaigns have addressed these challenges by utilizing behavioral science and customizing strategies for specific audiences, there are still gaps in understanding the factors that drive vaccination behavior. Socio-economic factors are insufficiently explored in the literature, particularly in terms of how economic status affects healthcare access and vaccine acceptance (13). Additionally, disparities between urban and rural vaccination rates require more investigation, as rural areas often face poorer healthcare infrastructure (14). The quality and design of public health campaigns are another area needing more study, particularly regarding which aspects most effectively change vaccination behavior (15). Lastly, logistical barriers, such as limited access to vaccination sites and lengthy waiting times, have not been thoroughly studied in terms of their impact on vaccination uptake (16). The role of public health campaigns in influencing vaccination behavior is widely recognized. The COVID-19 pandemic, for example has highlighted the importance of effective vaccination campaigns. It also, however revealed significant disparities in vaccine uptake influenced by various factors, including socio-economic status, geographic location, quality of public health information, mis-and dis-information and accessibility of vaccination services (1, 17).

**Figure 1:**
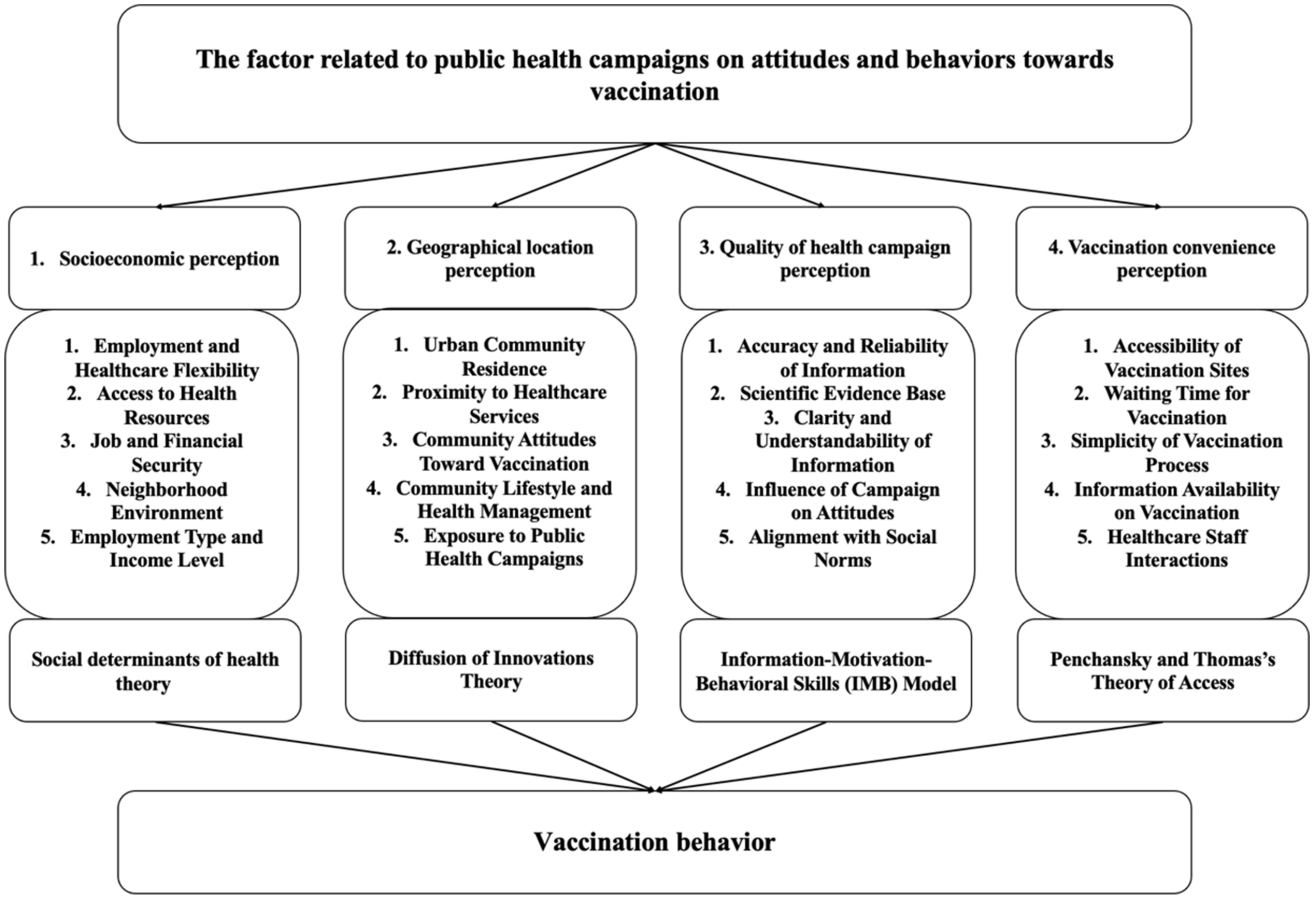
Theoretical framework of the research.

The correlation between individuals’ socioeconomic status and vaccination behaviors is well-documented, with factors such as income, education, and occupation playing a significant role. Higher socioeconomic status often leads to better healthcare access, health literacy, and trust in healthcare systems, which promotes vaccination uptake, while lower socioeconomic groups face barriers like misinformation and economic instability (18, 19). To address these disparities, policies aimed at reducing costs and improving access are effective (20). The Social Determinants of Health Theory explains how socioeconomic factors like income, education, employment security, and neighborhood conditions shape health behaviors, including vaccination (21). Geographic location also significantly impacts vaccination behaviors. Urban residents, with closer access to healthcare and public health campaigns, tend to have higher vaccination rates than rural residents, who face longer travel times and lower health literacy (22). The Diffusion of Innovations Theory suggests that urban populations are more likely to adopt new health interventions, while rural areas may adopt them more slowly due to limited resources and differing social norms (23). Public health campaign effectiveness is another critical factor, relying on the quality of messages, delivery methods, and cultural sensitivity. Campaigns that are clear, consistent, and address safety concerns are more successful, as explained by the Information-Motivation-Behavioral Skills (IMB) Model, which emphasizes providing accurate information, motivation, and access skills (24, 25). Convenience in accessing vaccination services, including the proximity of vaccination centers and ease of scheduling, plays a crucial role in improving vaccine uptake. The Theory of Access, by Penchansky and Thomas, highlights the importance of availability, accessibility, affordability, and acceptability of healthcare services (26, 30). These factors—socioeconomic status, geographical location, public health campaign quality, and convenience—form the theoretical framework of this study, guided by the Social Determinants of Health Theory, Diffusion of Innovations Theory, Information-Motivation-Behavioral Model, and Theory of Access, as reflected in the questionnaire designed for this research as shown in Figure 1.

The Wuxi region in China presents unique challenges in public health intervention activities due to its blend of urban and rural areas, diverse socioeconomic statuses, and varying public health infrastructures. In Wuxi, the disparities in vaccination behavior between urban and rural areas, as well as among different socio-economic groups, are particularly striking. While urban areas generally exhibit higher vaccination rates, rural areas are often left behind due to factors such as limited access to healthcare facilities, lower health literacy, and logistical challenges (27). In addition, individuals from lower socio-economic backgrounds in both urban and rural settings may have different perceptions and attitudes toward vaccination, influenced by their educational and economic statuses. Besides, factors such as the availability of vaccination centers, ease of scheduling appointments, and the efficiency of the vaccination process can significantly influence an individual’s decision to get vaccinated. This is particularly relevant in regions like Wuxi, where the distribution of healthcare resources and infrastructure is uneven.

Given these considerations, this study aims to examine the influence of selected factors related to public health campaigns on vaccination behavior, from the perspective of the targeted recipient population in the Wuxi region, China. By exploring these dynamics, the study seeks to contribute to the broader understanding of how public health strategies can be tailored to different contexts to improve vaccination uptake and, hopefully, the public health outcomes.

The central research question guiding this study is: “What are the factors related to public health campaigns that could influence the vaccination behavior among the population in the Wuxi region of China?” Specifically, the research questions are:

- What is the correlation between the socioeconomic status and vaccination behavior among the population of the Wuxi region, China?
- Are there any disparities in the vaccination behavior between the urban and rural population of the Wuxi region?
- What are the perceived characteristics of public health campaigns that can influence the vaccination behavior among the population?
- What are the perceived logistic factors that can affect the vaccination behavior of the population?

This leads to the following four hypotheses:

- Hypothesis 1 (H1): Individuals from higher socio-economic backgrounds are more likely to engage in vaccination behavior.
- Hypothesis 2 (H2): Urban residents are more likely to get vaccinated compared to rural residents.
- Hypothesis 3 (H3): Public health campaigns that are perceived as accurate, meticulous, and comprehensible have a more positive impact on vaccination behavior.
- Hypothesis 4 (H4): Greater convenience in accessing vaccination services (more vaccination sites, shorter waiting time, simpler vaccination process) leads to better vaccination behavior.

**Figure 2:**
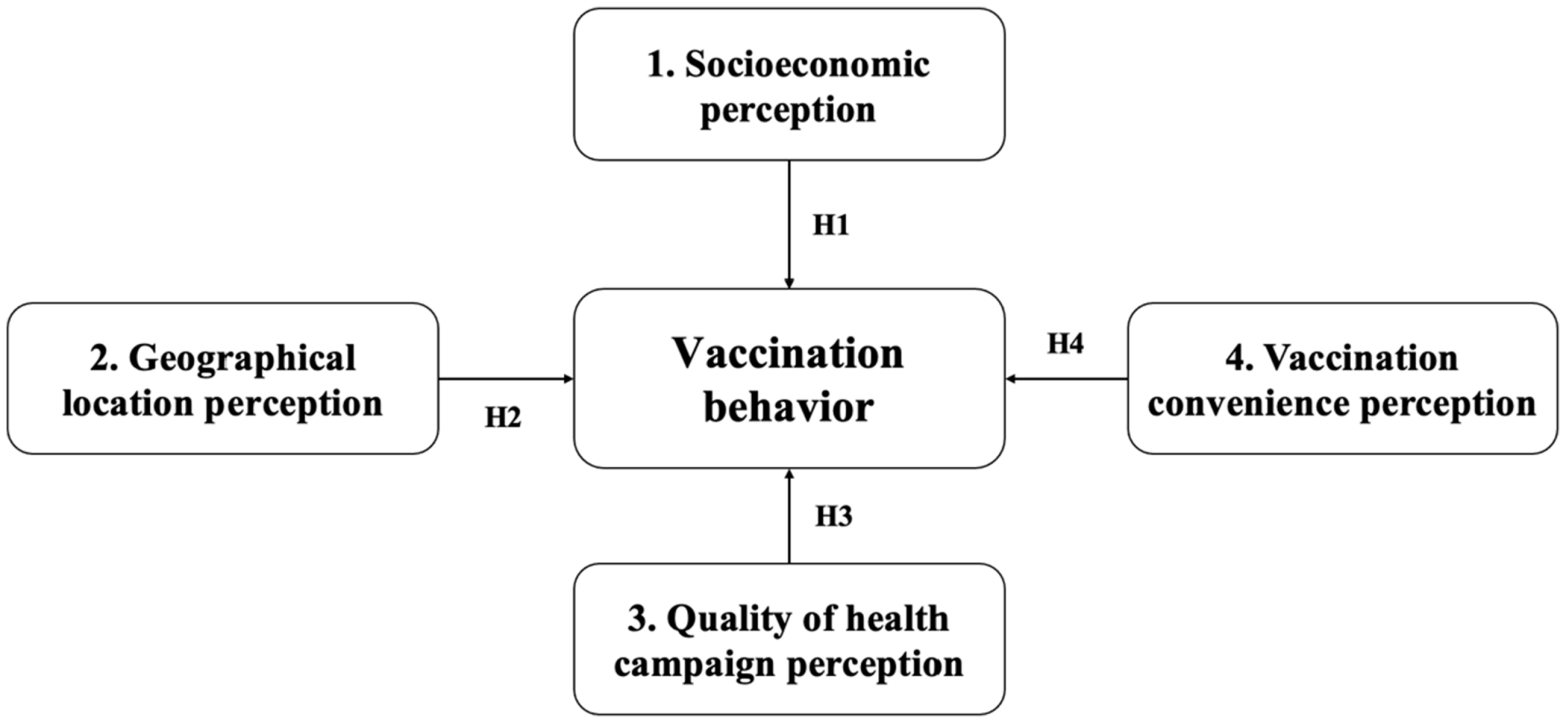
Research hypothesis diagram.

Figure 2 shows the research hypothesis diagram of this article, including independent variables, dependent variables, and corresponding hypotheses.

## 2 Methods

### 2.1 Overview

The study design is cross-sectional survey among the study participants sampled from the population of the Wuxi region, China. The information on the independent and dependent variables will be obtained at the same time using questionnaire.

### 2.2 Sample size, sampling strategy and data collection procedure

The sample size for this study was calculated using the method proposed by Hair et al. (28), applying a sample-to-variable ratio of 15:1 for exploratory factor analysis. With 35 independent variables, the initial sample size required was 525 participants. Considering a 30% non-response rate, the final sample size was increased to 750 respondents. The study employs a stratified random sampling method to ensure the sample reflects the population of Wuxi, Jiangsu, focusing on urban and rural distinctions. The population is divided into distinct strata, with each stratum sampled randomly to ensure equal selection chances. The primary strata are urban and rural residents, enabling the study to investigate differences in vaccination behavior between these groups.

Inclusion criteria for participants include being a resident of Wuxi, China, and aged 18 or older to ensure informed consent. Exclusion criteria consist of participants unable to read Mandarin Chinese or those refusing to participate and provide consent. Data was collected via an online self-administered questionnaire, distributed through the WeChat app or face-to-face interactions. The process was regularly monitored to ensure adequate response rates, with reminders sent every 2 weeks to participants who had not completed the questionnaire. After three reminders, non-respondents were excluded.

The questionnaire includes demographic information such as gender and education level, as well as independent variables: socioeconomic perception, geographical location perception, health campaign quality perception, and vaccination convenience perception. The dependent variable is vaccination behavior. The study first conducted a literature review to set measurement standards for public health campaigns and vaccination behavior. Guided by experts using the Delphi method, a scientific questionnaire was designed. Factor analysis then validated its structure and items, ensuring the quantitative measures met reliability and validity standards.

### 2.3 Statistical analysis

This study employs several statistical analyses, executed using both SPSS and R software, to explore the relationships between socioeconomic, geographic, and health campaign factors with vaccination behavior. First, descriptive statistics are used to summarize demographic variables, including age, gender, education, marital status, number of children, occupation, field of work, and type of residence.

Next, a reliability and validity analysis are conducted to assess the internal consistency and accuracy of the measurement instruments. Cronbach’s alpha is used to evaluate the reliability, ensuring the stability and consistency of the scales measuring constructs such as socioeconomic perception, geographical location perception, quality of health campaigns, and vaccination convenience. Kaiser-Meyer-Olkin (KMO) and factor analysis are applied to assess the suitability of the data for exploratory factor analysis, confirming the validity of the constructs.

Following this, correlation analysis is performed to examine the linear relationships between the independent variables (socioeconomic perception, geographical location perception, quality of health campaigns, and vaccination convenience) and the dependent variable (vaccination behavior). Pearson’s correlation coefficient is used to quantify the strength and direction of these relationships, providing insight into how these factors influence vaccination behavior.

Finally, regression analysis is employed to determine the predictive power of the independent variables on vaccination behavior. Multiple regression models are developed to assess the effects of socioeconomic status, geographic location, quality of health campaigns, and vaccination convenience on three aspects of vaccination behavior: willingness to receive vaccines, actual vaccinations after the age of 18, and future vaccination intentions. This analysis helps to identify the significant predictors of vaccination behavior, with a focus on the influence of accessibility, public health campaign quality, and socioeconomic and geographic factors.

## 3 Results

### 3.1 Descriptive statistics

**Figure 3:**
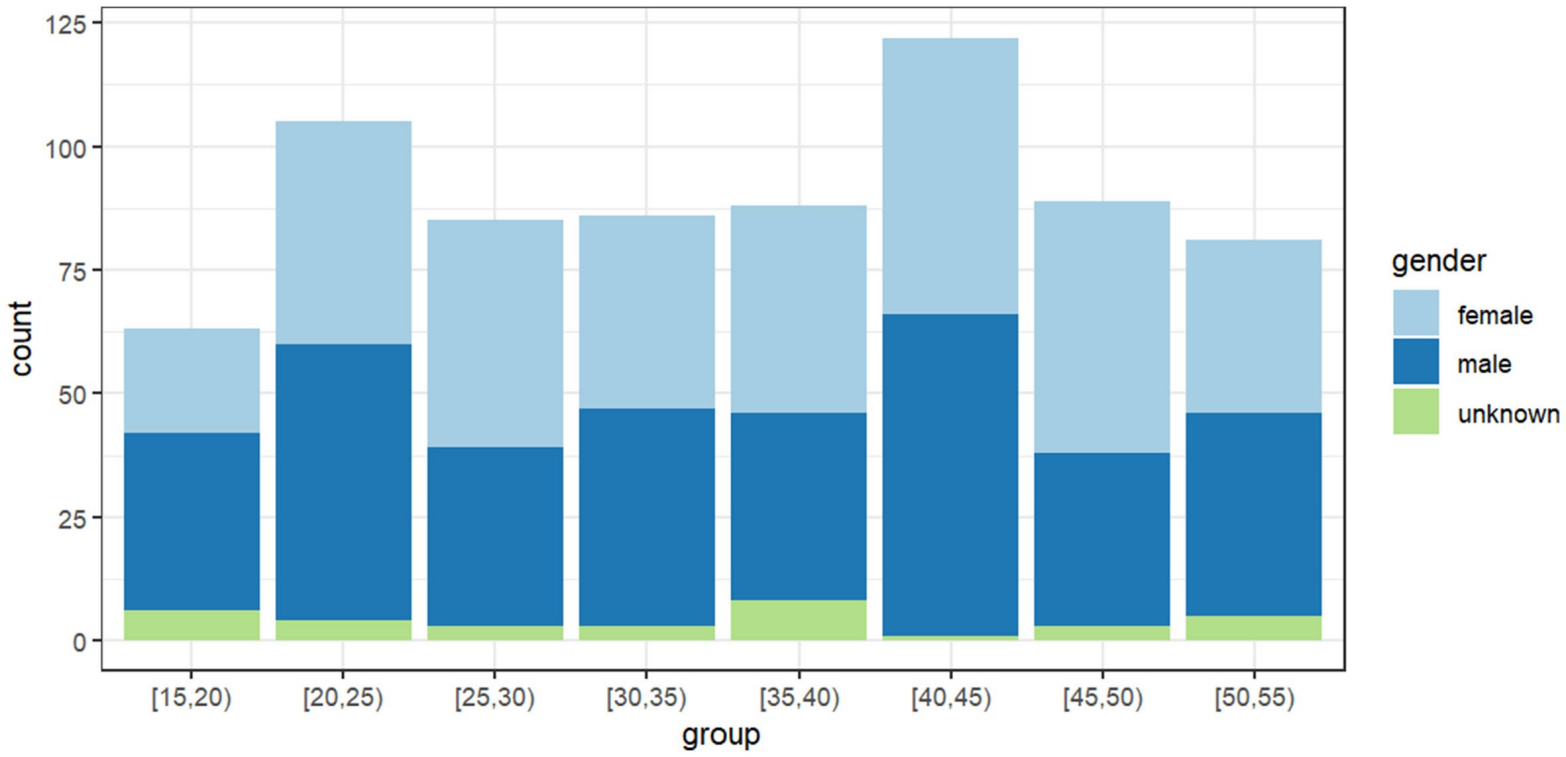
The gender count among different age groups.

**Figure 4:**
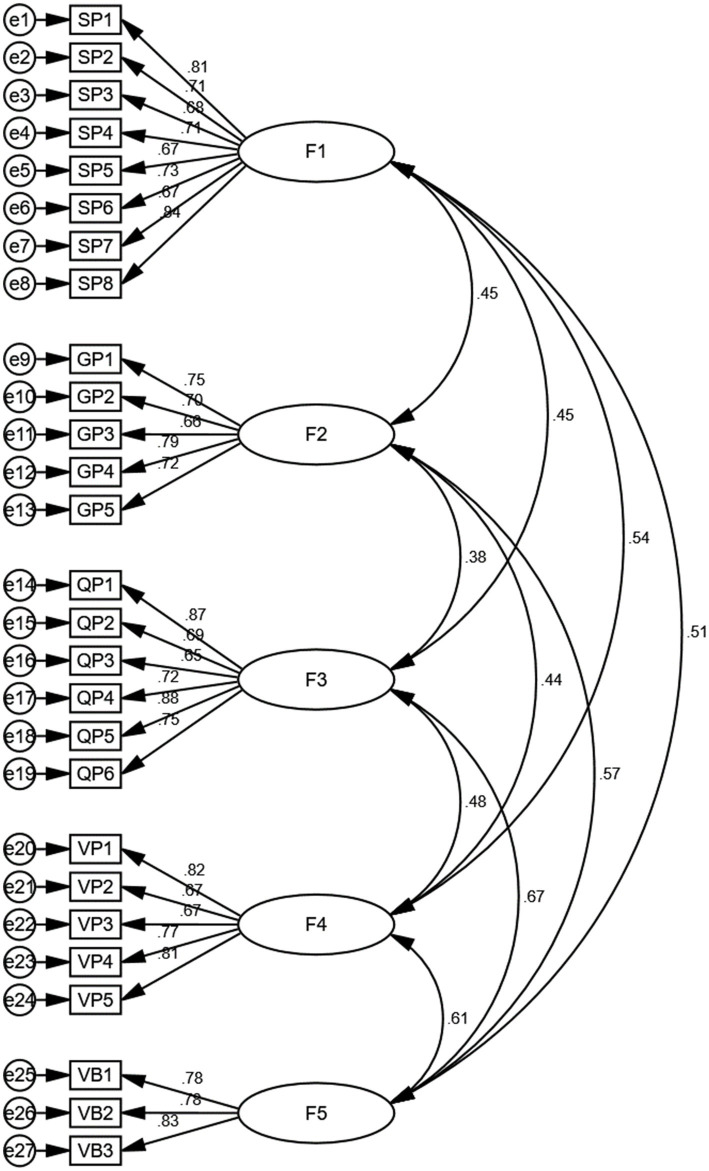
Path diagram of a structural equation model illustrating hypothesized relationships. F1: Socioeconomic perception, F2: Geographical location perception, F3: Quality of health campaign perception, F4: Vaccination convenience perception, F5: Vaccination behavior.

The gender distribution of the respondents reveals a fairly balanced composition with a slight male predominance. Out of 719 participants, 351 (48.8%) are male, 335 (46.6%) are female, and 33 (4.6%) preferred not to disclose their gender. The mean value of the age is 35.1, and the median is 36, while the standard deviation is 10.8. The age distribution is relatively even, but it is more prominent in groups [20, 25) and [40, 45). According to Figure 3, there is no significant difference between the males and females (Figure 4).

Education levels among the participants vary significantly, ranging from primary school to doctoral degrees. The majority of respondents hold a bachelor’s degree (33.1%), followed by senior high school graduates (29.8%) and junior high school graduates (16.8%). Participants with a master’s degree constitute 14.7%, while those with primary school education, doctoral degrees, or preferring not to disclose their education level represent a smaller fraction (2.6, 1.8, and 1.1% respectively).

Marital status data shows that 44.1% of respondents are single, 36.0% are married, 10.0% are divorced, 1.9% are widowed, and 7.9% preferred not to disclose their marital status.

The number of children per respondent also shows notable variability. A significant portion of the participants (61.6%) reported having no children, while 31.3% have one child, 6.8% have two children, and a minimal 0.3% have three children.

Occupation data reveals a diverse range of professional backgrounds. The largest groups are students (27.4%) and government or public service employees (22.1%), followed by private sector employees (non-healthcare) at 18.6%, and self-employed individuals at 10.7%. Smaller groups include healthcare professionals (3.2%), educators (4.7%), unemployed individuals (6.8%), retired individuals (2.1%), and those specifying other occupations (4.3%).

Field of work further diversifies the respondent pool with notable representations from various sectors. Students again form the largest group (26.8%), followed by individuals in engineering and technology (13.6%), government or public administration (13.5%), business and finance (12.1%), and non-profit/community service (7.2%). Other sectors include healthcare and medical services (4.0%), education and academic research (4.9%), arts and entertainment (4.0%), and unemployed/retired individuals (9.7%).

Type of residence data shows that the majority of respondents reside in single-family homes (42.8%) and apartments/condominiums (34.8%). Shared housing, such as dormitories or roommate situations, accounts for 15.6%, while assisted living facilities and temporary housing constitute smaller portions (4.0 and 2.8% respectively).

Table 1 presents the sociodemographic characteristics of the respondents. Overall, the respondents show diversity in terms of gender, education level, marital status, number of children, occupation, field of work, and type of residence. Among them, the education level is mainly dominated by bachelor’s degree holders, marital status is primarily single, the majority of respondents have no children, and occupation and field of work cover a wide range. The type of residence is mainly single-family homes or apartments.

**Table 1:** Sociodemographic characteristics of the respondents.

Variable	*N*	Mean	Standard deviation
Gender	719	1.5577	0.58218
Education	719	3.5035	1.13303
Marital status	719	1.936	1.15333
Number of children	719	1.4576	0.63384
Occupation	719	4.4395	1.78945
Field of work	719	5.975	2.49276
Type of resident	719	1.8915	0.99269

Table 2 the chi-square test results reveal various insights across different sociodemographic variables. For gender, the Pearson Chi-Square value is 32.762 with a *p*-value of 0.109, indicating no significant association. However, for education, there is a significant association with a Pearson Chi-Square value of 98.145 and a *p*-value of 0.022, supported by the Likelihood Ratio (*p* = 0.005) and Linear-by-Linear Association (*p* = 0.002). Marital status and number of children both show no significant associations with *p*-values of 0.612 and 0.835, respectively. Occupation reveals a marginally significant association (*p* = 0.04), though the Likelihood Ratio (*p* = 0.14) suggests weaker evidence. Field of work presents a significant association (*p* = 0.013), supported by the Likelihood Ratio (*p* = 0.007) and the Linear-by-Linear Association (*p* = 0.004). Finally, type of residence shows no significant association, with a Pearson Chi-Square *p*-value of 0.32. Overall, education and field of work appear to have the strongest associations with other variables.

**Table 2:** Differences in demographic factors generate differences in vaccination behavior.

	Chi-square test	Value	Degrees of freedom	Asymptotic significance (two-sided)
Gender	Pearson Chi-square	32.762a	24	0.109
	Likelihood ratio	33.212	24	0.1
	Linear-by-linear association	3.894	1	0.048
	Number of valid cases	719		
Education	Pearson chi-square	98.145	72	0.022
	Likelihood ratio	106.202	72	0.005
	Linear-by-linear association	9.55	1	0.002
	Number of valid cases	719		
Marital status	Pearson chi-square	13.816	16	0.612
	Likelihood ratio	16.11	16	0.445
	Linear-by-linear association	1.289	1	0.256
	Number of valid cases	719		
Number of children	Pearson chi-square	7.336	12	0.835
	Likelihood ratio	8.364	12	0.756
	Linear-by-linear association	0.155	1	0.694
	Number of valid cases	719		
Occupation	Pearson chi-square	121.509	96	0.04
	Likelihood ratio	111.059	96	0.14
	Linear-by-linear association	1.796	1	0.18
	Number of valid cases	719		
Field of work	Pearson chi-square	57.518	36	0.013
	Likelihood ratio	60.043	36	0.007
	Linear-by-linear association	8.231	1	0.004
	Number of valid cases	719		
Type of resident	Pearson chi-square	18.067	16	0.32
	Likelihood ratio	19.014	16	0.268
	Linear-by-linear association	0.595	1	0.44
	Number of valid cases	719		

#### 3.1.1 Socioeconomic perception and vaccination behavior

In addition to demographic factors, the perception of health campaign quality also plays a crucial role in influencing vaccination behavior. Understanding how individuals perceive the effectiveness and trustworthiness of health campaigns can shed light on their willingness to get vaccinated. A positive perception of health campaigns may enhance public trust and acceptance of vaccines, thereby improving vaccination rates across different demographic groups. This underscores the importance of effective communication strategies in health campaigns aimed at increasing vaccination uptake. An example of the impact of health campaign quality can be seen in the HPV vaccination initiatives in several countries. In Australia, a well-structured health campaign was launched to promote the HPV vaccine among adolescents. The campaign utilized clear messaging, targeted outreach, and endorsements from trusted healthcare professionals. As a result, vaccination rates among teenage girls rose significantly from around 20% to over 80% within a few years. The campaign’s success was attributed to its high perceived quality, which effectively addressed concerns and misinformation about the vaccine.

#### 3.1.2 Geographical location perception and vaccination behavior

The geographical location perception section of the questionnaire mainly assesses participants’ views on their living environment (urban or rural) and the accessibility of healthcare resources. This perception may influence the availability of vaccination services and individuals’ willingness to get vaccinated. The survey includes evaluations of the distance to vaccination sites, transportation convenience, and healthcare service quality. By comparing vaccination behaviors under different geographical location perceptions, the study seeks to identify disparities between urban and rural residents in terms of vaccine access and decision-making, providing a basis for optimizing vaccination strategies in various regions.

#### 3.1.3 Perception of health campaign quality and vaccination behavior

Health campaigns that are perceived as high-quality, credible, and informative are likely to foster better vaccination behaviors. Factors contributing to the perception of campaign quality include message clarity, delivery methods, and the perceived expertise of health authorities. Tailoring these campaigns to address specific demographic concerns and preferences can further enhance their effectiveness. As such, continuous assessment of public perceptions regarding health campaigns is vital for refining strategies to promote vaccination and improve overall public health outcomes. In the UK, there was a notable decline in MMR vaccination rates following the publication of a controversial study linking the vaccine to autism. In response, the National Health Service (NHS) launched a comprehensive campaign to restore public trust and encourage vaccination. The campaign emphasized transparency, addressed specific parental concerns, and utilized multiple platforms, including social media, community outreach, and partnerships with healthcare providers. As a result, MMR vaccination rates rose significantly over the following years, with uptake returning to pre-scandal levels. This case demonstrates how effectively addressing public perceptions and concerns can lead to improved vaccination behavior.

#### 3.1.4 Perception of vaccination convenience and vaccination behavior

The vaccination convenience perception section of the questionnaire primarily measures participants’ views on the ease of the vaccination process, including appointment procedures, distance to vaccination sites, waiting times, and service availability. The convenience of vaccination is a key factor influencing vaccination behavior. By analyzing the relationship between convenience perception and vaccination behavior, the study seeks to identify areas for optimizing vaccination services and provide empirical evidence for strategies aimed at increasing vaccination rates. Higher convenience perception is generally associated with greater willingness to get vaccinated, making the improvement of vaccination processes a critical approach to boosting vaccination coverage.

Table 3 presents the descriptive statistics of the independent variables based on a sample size of 719 participants. The mean score for socioeconomic perception is 3.1219, with a standard deviation of 0.83062, indicating moderate perceptions of socioeconomic status with some variability among respondents. The geographical location perception has a higher mean of 3.8503 and a standard deviation of 0.80636, suggesting that most participants view their geographical location more favorably in terms of healthcare accessibility. The quality of health campaign perception also has a relatively high mean of 3.8389 and a standard deviation of 0.82964, reflecting generally positive views on the effectiveness of health promotion efforts. Lastly, the vaccination convenience perception has a mean of 3.3994 and a standard deviation of 0.77883, indicating a moderate level of perceived convenience regarding the vaccination process (Tables 4–11).

**Table 3:** Description of the independent variable statistics.

Variable	*N*	Mean	Standard deviation
Socioeconomic perception	719	3.1219	0.83062
Geographical location perception	719	3.8503	0.80636
Quality of health campaign perception	719	3.8389	0.82964
Vaccination convenience perception	719	3.3994	0.77883

**Table 4:** Vaccination rates by urban and rural residency.

Location	Vaccination rate
Rural	93.75%
Urban	96.87%

**Table 5:** Vaccination rates by educational level.

Education level	Vaccination
Primary school	94.74%
Junior high school	94.21%
Senior high school	97.20%
Bachelor degree	97.06%
Master degree	99.06%
Doctoral degree or higher	99.00%

**Table 6:** Vaccination rates by age group.

Age group	Vaccination
(0, 18]	95.45%
(18, 30]	98.25%
(30, 50]	95.83%
(50, 100]	98.39%

**Table 7:** Reliability analysis and KMO test.

Variables	Cronbach’s *α*	KMO^*^	*N*
Socioeconomic perception	0.899	0.916	8
Geographical location perception	0.844	0.853	5
Quality of health campaign perception	0.888	0.907	6
Vaccination convenience perception	0.860	0.870	5
Vaccination behavior	0.837	0.721	3

* KMO, Kaiser-Meyer-Olkin values.

**Table 8:** Validity analysis.

Variables	Measurement item	Factor loading	Cronbach’s *α*	CR^*^	AVE^*^
F1: Socioeconomic perception	SP1	0.815	0.899	0.901	0.534
	SP2	0.707			
	SP3	0.675			
	SP4	0.712			
	SP5	0.673			
	SP6	0.734			
	SP7	0.671			
	SP8	0.837			
F2: Geographical location perception	GP1	0.746	0.844	0.847	0.525
	GP2	0.699			
	GP3	0.663			
	GP4	0.787			
	GP5	0.722			
F3: Quality of health campaign perception	QP1	0.874	0.888	0.894	0.587
	QP2	0.695			
	QP3	0.655			
	QP4	0.720			
	QP5	0.875			
	QP6	0.749			
F4: Vaccination convenience perception	VP1	0.820	0.860	0.864	0.863
	VP2	0.666			
	VP3	0.666			
	VP4	0.775			
	VP5	0.809			
F5: Vaccination behavior	VB1	0.781	0.837	0.840	0.636
	VB2	0.776			
	VB3	0.834			

* CR, composite reliability; AVE, average variance extracted.

**Table 9:** The summary result of VB3.

Significant variables for VB3	Future vaccination intentions			
	S.E.	*β*	*t*	*p*
SP	0.027	0.235**	8.585	<0.0001
GP	0.032	0.221***	6.933	<0.0001
QP	0.036	0.194***	5.408	<0.0001
VP	0.029	0.237***	8.058	<0.0001
*R^2^*	0.4369			
Δ*R*^2^	0.4337			
*F*	138.5***			

***p < 0.001, **p < 0.01, *p < 0.05.

**Table 10:** The summary result of VB1.

Significant variables for VB1	Willingness to receive vaccine			
	S.E.	*β*	*t*	*p*
SP	0.030	0.238**	7.878	<0.0001
GP	0.035	0.255***	7.252	<0.0001
QP	0.040	0.185***	4.676	<0.0001
VP	0.032	0.202***	6.259	<0.0001
*R^2^*	0.3898			
Δ*R*^2^	0.3864			
*F*	114***			

***p < 0.001, **p < 0.01, *p < 0.05.

**Table 11:** The summary result of VB2.

Significant variables for VB2	Number of vaccines received			
	S.E.	*β*	*t*	*p*
SP	0.030	0.296***	9.788	<0.0001
GP	0.035	0.172***	4.876	<0.0001
QP	0.040	0.206***	5.173	<0.0001
VP	0.032	0.256***	7.882	<0.0001
*R^2^*	0.4132			
Δ*R*^2^	0.4091			
*F*	100.4***			

***p < 0.001, **p < 0.01, *p < 0.05.

Vaccination rates differ between urban and rural areas, with urban residents showing a higher vaccination rate of 96.87% compared to 93.75% for rural residents. This discrepancy can be attributed to several factors. Urban residents often have better access to information regarding vaccination policies and benefits, coupled with superior medical infrastructure, making vaccination more accessible. Furthermore, urban populations may exhibit a stronger vaccination awareness due to higher levels of education and exposure to health campaigns. Conversely, rural areas may face challenges such as limited healthcare resources, logistical issues, and lower vaccine awareness, all of which contribute to the lower vaccination rates.

Educational attainment demonstrates a strong positive correlation with vaccination rates. Individuals with primary school education exhibit the lowest vaccination rate at approximately 94.74%, while those with advanced degrees (Master’s and Doctoral levels) have rates near universal vaccination, at 99.06 and 99%, respectively. This trend highlights the pivotal role of education in promoting vaccine uptake. Higher education levels enhance individuals’ ability to comprehend the scientific basis and importance of vaccines, leading to increased adherence. Additionally, individuals with advanced education are more likely to prioritize health and demonstrate greater compliance with public health policies. These findings suggest that targeted educational campaigns for populations with lower educational attainment could significantly boost overall vaccination coverage.

Vaccination rates vary across age groups, with a notable trend of higher uptake among middle-aged individuals compared to younger or older age groups. The 18 and under cohort exhibits lower vaccination rates, potentially due to parental hesitancy or policy restrictions for minors. In contrast, the 18–30 age group shows a significant increase, likely driven by higher educational attainment and frequent social interactions. The 30–50 age group achieves the highest vaccination rates, reflecting their heightened health awareness and societal responsibilities during prime working years. However, rates slightly decline in the 50 and older group, possibly due to vaccine safety concerns or pre-existing health conditions. These findings underscore the need for tailored vaccination strategies, such as enhancing safety communication for older adults and engaging parents of minors to address hesitancy effectively.

### 3.2 Reliability and validity analysis

The reliability and validity of the questionnaires used in this study are critical for ensuring the consistency, stability, and accuracy of the measurement of the predetermined concepts. A Cronbach’s *α* value greater than 0.7 is generally considered acceptable, while values above 0.8 indicate good reliability, and those above 0.9 signify excellent reliability.

For the socioeconomic perception variable, the Cronbach’s α coefficient is 0.899, which indicates excellent reliability. This high value demonstrates that the items measuring socioeconomic perception are consistently reflecting the underlying construct. The Kaiser-Meyer-Olkin (KMO) measure of sampling adequacy is 0.916, which is well above the acceptable threshold of 0.5, suggesting that the data is suitable for factor analysis. The number of items (*N*) included in this variable is eight, further supporting the robustness of the reliability assessment.

The geographical location perception variable has a Cronbach’s *α* coefficient of 0.844, indicating good reliability. The KMO measure for this variable is 0.853, which also supports the suitability of the data for factor analysis. With five items included in this variable, the reliability analysis confirms that the items are consistently measuring the intended construct.

The quality of health campaign perception variable demonstrates a Cronbach’s *α* coefficient of 0.888, indicating excellent reliability. The KMO measure for this variable is 0.907, which is high and confirms the appropriateness of the data for factor analysis. This variable includes six items, ensuring a comprehensive assessment of the quality of health campaigns.

The vaccination convenience perception variable has a Cronbach’s *α* coefficient of 0.860, reflecting excellent reliability. The KMO measure for this variable is 0.870, indicating that the data is suitable for factor analysis. With five items included in this variable, the reliability analysis shows that the items are consistently measuring the perception of vaccination convenience.

The dependent variable, vaccination behavior, has a Cronbach’s *α* coefficient of 0.837, indicating good reliability. The KMO measure for this variable is 0.721, which is acceptable and supports the suitability of the data for factor analysis. This variable includes three items, demonstrating that the items are reliably measuring vaccination behavior.

Construct validity is evaluated using factor loading, composite reliability (CR), and average variance extracted (AVE). High factor loadings (greater than 0.6) indicate that the items are well-correlated with the latent construct they are intended to measure. Composite reliability (CR) values greater than 0.7 are considered acceptable, while AVE values above 0.5 indicate good convergent validity.

For the socioeconomic perception variable, the factor loadings for the eight measurement items range from 0.671 to 0.837, demonstrating strong correlations with the latent construct. The Cronbach’s *α* coefficient is 0.899, the CR is 0.901, and the AVE is 0.534, all of which indicate excellent construct validity. These results confirm that the items accurately measure the socioeconomic perception construct.

The geographical location perception variable shows factor loadings ranging from 0.663 to 0.787, indicating good correlations with the latent construct. The Cronbach’s *α* coefficient is 0.844, the CR is 0.847, and the AVE is 0.525, which demonstrate good construct validity. These findings confirm that the items effectively measure the geographical location perception construct.

For the quality of health campaign perception variable, the factor loadings for the six measurement items range from 0.655 to 0.875, with the majority above 0.7, indicating strong correlations with the latent construct. The Cronbach’s *α* coefficient is 0.888, the CR is 0.894, and the AVE is 0.587, all of which indicate excellent construct validity. These results validate that the items accurately measure the quality of health campaign perception construct.

The vaccination convenience perception variable shows factor loadings ranging from 0.666 to 0.820, indicating strong correlations with the latent construct. The Cronbach’s *α* coefficient is 0.860, the CR is 0.864, and the AVE is 0.863, which demonstrate excellent construct validity. These findings confirm that the items effectively measure the vaccination convenience perception construct.

Finally, the vaccination behavior variable has factor loadings ranging from 0.776 to 0.834, indicating strong correlations with the latent construct. The Cronbach’s *α* coefficient is 0.837, the CR is 0.840, and the AVE is 0.636, all of which indicate good construct validity. These results validate that the items accurately measure the vaccination behavior construct.

The reliability and validity analyses of the questionnaires demonstrate that the measurement instruments used in this study are both consistent and accurate in measuring the intended constructs. The high Cronbach’s *α* coefficients and KMO measures confirm the internal consistency and suitability of the data for factor analysis, while the strong factor loadings, composite reliability, and average variance extracted values validate the construct validity of the questionnaires.

### 3.3 Correlation analysis

The correlation analysis provides critical insights into the linear relationships between socioeconomic perception, geographical location perception, quality of health campaign perception, vaccination convenience perception, and vaccination behavior, thereby elucidating the strength and direction of these relationships using the confirmatory factor analysis via structural equation modeling.

#### 3.3.1 Socioeconomic perception and other variables

The structural equation modeling analysis found that the correlation between socioeconomic perception and geographical location perception is 0.45, indicating a moderate positive linear relationship. This suggests that individuals with a higher socioeconomic perception are more likely to have a positive perception of their geographical location.

The correlation between socioeconomic perception and quality of health campaign perception is 0.45, also indicating a moderate positive linear relationship. This implies that individuals who perceive their socioeconomic status favorably are likely to have a positive perception of the quality of health campaigns.

Additionally, the correlation between socioeconomic perception and vaccination convenience perception is 0.54, indicating a strong positive linear relationship. This suggests that individuals with a higher socioeconomic perception tend to perceive vaccination as more convenient.

Lastly, the correlation between socioeconomic perception and vaccination behavior is 0.51, indicating a moderate positive linear relationship. This means that individuals with a favorable perception of their socioeconomic status are more likely to exhibit positive vaccination behavior.

#### 3.3.2 Geographical location perception and other variables

The correlation between geographical location perception and quality of health campaign perception is 0.38, indicating a moderate positive linear relationship. This suggests that individuals who perceive their geographical location positively are likely to have a favorable perception of the quality of health campaigns.

The correlation between geographical location perception and vaccination convenience perception is 0.44, indicating a moderate positive linear relationship. This implies that individuals who perceive their geographical location positively tend to perceive vaccination as more convenient.

Moreover, the correlation between geographical location perception and vaccination behavior is 0.57, indicating a strong positive linear relationship. This suggests that individuals with a favorable perception of their geographical location are more likely to exhibit positive vaccination behavior.

#### 3.3.3 Quality of health campaign perception and other variables

The correlation between quality of health campaign perception and vaccination convenience perception is 0.48, indicating a strong positive linear relationship. This implies that individuals who perceive the quality of health campaigns positively are likely to perceive vaccination as more convenient.

Furthermore, the correlation between quality of health campaign perception and vaccination behavior is 0.67, indicating a very strong positive linear relationship. This suggests that individuals with a favorable perception of the quality of health campaigns are highly likely to exhibit positive vaccination behavior.

#### 3.3.4 Vaccination convenience perception and vaccination behavior

The correlation between vaccination convenience perception and vaccination behavior is 0.61, indicating a strong positive linear relationship. This means that individuals who perceive vaccination as convenient are more likely to exhibit positive vaccination behavior.

#### 3.3.5 Summary of correlation analysis

The correlation analysis reveals significant positive linear relationships between the variables under study. The moderate to strong positive correlations between socioeconomic perception, geographical location perception, quality of health campaign perception, and vaccination convenience perception with vaccination behavior suggest that these independent variables are likely to influence vaccination behavior positively.

Specifically, the strongest correlation is observed between quality of health campaign perception and vaccination behavior, indicating that the perceived quality of health campaigns plays a crucial role in shaping vaccination behavior. This finding underscores the importance of delivering high-quality health campaigns to promote positive vaccination behavior among the population.

The strong positive correlation between vaccination convenience perception and vaccination behavior highlights the significance of making vaccination services accessible and convenient to encourage higher vaccination rates.

The moderate positive correlations between socioeconomic perception, geographical location perception, and vaccination behavior suggest that improving perceptions of socioeconomic status and geographical location can also positively impact vaccination behavior.

These findings provide a robust basis for hypothesis testing and causal inference in subsequent analyses. The positive linear relationships between the independent variables and vaccination behavior indicate that enhancing socioeconomic perceptions, geographical location perceptions, the quality of health campaigns, and vaccination convenience can potentially lead to improved vaccination behavior. This correlation analysis, therefore, forms a crucial component of understanding the impact of public health campaigns on attitudes and behaviors toward vaccination, guiding future interventions and policy decisions to enhance vaccination uptake.

### 3.4 Regression analysis

The results from the regression analysis provide significant insights into the factors influencing vaccination behavior among a sample of 719 individuals. The regression model includes several predictors such as the level of education, field of work, perceptions related to vaccination convenience, quality of health campaign, geographical location, and socioeconomic status.

The regression analysis conducted for VB1, VB2, and VB3 reveals important insights into the relationship between socio-economic factors, urban residency, public health campaign quality, and accessibility to vaccination services on vaccination behavior. The dependent variable in each case represents a dimension of vaccination behavior, including willingness to receive vaccines recommended by public health campaigns (VB1), the actual number of vaccines received after the age of 18 (VB2), and future vaccination intentions (VB3). The independent variables—socio-economic position (SP), geographic location (GP), the quality of public health campaigns (QP), and the accessibility of vaccination services (VP)—serve as predictors in each regression model.

For VB1, the significant predictors were SP (*β* = 0.238, *p* < 0.01), GP (*β* = 0.255, *p* < 0.001), QP (*β* = 0.185, *p* < 0.001), and VP (*β* = 0.202, *p* < 0.001), with an *R*^2^ value of 0.3898, indicating that approximately 39% of the variance in willingness to receive vaccines could be explained by the independent variables. Among these, GP showed the highest contribution to vaccination willingness, with a standardized *β* value of 0.255. This indicates that urban residency plays a strong role in influencing willingness to get vaccinated. The high *β* values for both SP and VP suggest that socio-economic status and accessibility to vaccination sites also significantly influence the decision-making process. These results support Hypotheses 1, 2, and 4, confirming that individuals from higher socio-economic backgrounds and those living in urban areas are more likely to engage in vaccination behavior, while better accessibility to vaccination services enhances this behavior as well. The contribution of QP, though significant, was slightly lower than that of SP, GP, and VP, indicating that while the quality of public health campaigns matters, it may not be as influential as socio-economic or geographic factors.

In the case of VB2, the dependent variable, which measured the number of vaccinations received after the age of 18, was again predicted by SP (*β* = 0.296, *p* < 0.001), GP (*β* = 0.172, *p* < 0.001), QP (*β* = 0.206, *p* < 0.001), and VP (*β* = 0.256, *p* < 0.001), with an *R*^2^ value of 0.4132, suggesting that 41.32% of the variance in actual vaccination behavior could be explained by these predictors. Interestingly, SP contributed the most to VB2, with a *β* value of 0.296, implying that socio-economic factors have a greater influence on actual vaccination rates than they do on mere willingness. This finding lends further support to Hypothesis 1, demonstrating that individuals from higher socio-economic backgrounds not only express greater willingness but also follow through with actual vaccinations more frequently. VP also showed a strong effect (*β* = 0.256), suggesting that convenience and accessibility play a substantial role in determining how often individuals get vaccinated, which supports Hypothesis 4. The relatively lower contribution of GP (*β* = 0.172) compared to VB1 might suggest that geographic location is less influential when it comes to actual behavior than it is for willingness. The consistent performance of QP across both VB1 and VB2 highlights that well-executed public health campaigns do indeed result in higher vaccination rates, supporting Hypothesis 3.

For VB3, the dependent variable measured future vaccination intentions, and the regression results showed that all four predictors were significant once again: SP (*β* = 0.235, *p* < 0.01), GP (*β* = 0.221, *p* < 0.001), QP (*β* = 0.194, *p* < 0.001), and VP (*β* = 0.237, *p* < 0.001), with an *R*^2^ value of 0.4369, indicating that 43.69% of the variance in future vaccination intentions was explained by these variables. In this case, VP had the highest contribution (*β* = 0.237), followed closely by SP (*β* = 0.235) and GP (*β* = 0.221). This suggests that in terms of future vaccination behavior, accessibility and socio-economic factors are almost equally influential. Hypotheses 1 and 4 are strongly supported here, showing that individuals from higher socio-economic backgrounds, as well as those with better access to vaccination services, are more likely to plan to adhere to future vaccination recommendations. The influence of GP was also relatively strong in this model, demonstrating that urban residents continue to show a higher likelihood of future vaccination adherence, thus supporting Hypothesis 2. The lower yet still significant effect of QP (*β* = 0.194) indicates that while public health campaigns are important, their influence on future vaccination plans may not be as pronounced as the direct impact of accessibility or socio-economic position.

The results of the regression analysis across all three models—VB1, VB2, and VB3—consistently support the four proposed hypotheses, revealing clear causal relationships between the independent variables and vaccination behavior. Socio-economic position (SP) is shown to have a strong influence on both actual vaccination rates and future intentions, confirming that individuals from wealthier backgrounds are more likely to engage in vaccination behavior. This could be attributed to better access to information, higher trust in medical institutions, and greater resources to prioritize health-related activities. Geographic location (GP) also proves to be a significant predictor, with urban residents demonstrating higher vaccination rates and intentions, likely due to better access to healthcare facilities, more exposure to public health campaigns, and greater convenience in receiving vaccinations.

The contribution of public health campaign quality (QP) suggests that the accuracy, comprehensibility, and thoroughness of public messaging play a crucial role in influencing vaccination behavior, though its impact is somewhat less than socio-economic or accessibility factors. Finally, the accessibility of vaccination services (VP) emerges as a critical determinant across all three models, underscoring the importance of removing logistical barriers to vaccination in order to improve both current and future vaccination behavior. Making vaccinations more accessible by increasing the number of sites, reducing waiting times, and simplifying the process will likely result in higher vaccination rates and better public health outcomes overall.

In conclusion, this regression analysis validates the theoretical assumptions outlined in the four hypotheses. It reveals that socio-economic position, geographic location, public health campaign quality, and accessibility all contribute significantly to vaccination behavior, with socio-economic factors and accessibility emerging as particularly influential. These findings not only provide valuable empirical support for public health initiatives but also offer practical insights for policymakers aiming to improve vaccination rates by addressing socio-economic disparities, enhancing campaign quality, and removing barriers to accessibility.

## 4 Discussion

### 4.1 Strategies based on socio-economic status

To bridge the vaccination gap across socio-economic statuses, it is essential to develop targeted strategies that cater specifically to the needs of lower socio-economic groups. These strategies should focus on eliminating barriers such as financial constraints, access to reliable information, and healthcare resources. Programs offering financial aid, such as vaccine subsidies and transport vouchers, can alleviate some of the economic burdens. Information dissemination efforts should be intensified through community outreach and the use of digital platforms to ensure that correct and accessible information reaches all demographic segments. Additionally, the establishment of mobile vaccination clinics can enhance access by delivering services directly to under-served areas, making it easier for residents to get vaccinated without the need to travel far. Collaborations with local community organizations can further promote vaccination through trusted networks, providing a holistic approach to increasing vaccination rates among vulnerable populations.

### 4.2 Geographical-based strategies

Addressing the unique challenges faced by rural areas requires specific strategies that focus on logistical barriers and access to healthcare. Increasing the number of mobile vaccination units and temporary sites can substantially improve accessibility for rural residents, ensuring that vaccinations are available closer to home. Partnerships with local organizations can provide logistical support and facilitate the identification of strategic locations for these mobile units and sites. Additionally, leveraging technology for better scheduling and information dissemination can optimize the vaccination process, making it more efficient and accessible. Engaging with local leaders and influencers to promote vaccination benefits can also play a crucial role in building trust and encouraging community participation in vaccination programs.

### 4.3 Enhancing the effectiveness of public health campaigns

Research has shown that clear and precise messaging is critical for public understanding and behavior change. For instance, a study by Motta et al. (29) found that campaigns with regularly updated information maintained higher trust and engagement levels among the target audience. Ensuring the reliability of campaign content through expert reviews and regular updates is crucial. Campaigns should also aim to be understandable to people of all literacy levels by using simple language and visual aids, which help in explaining complex health information. Utilizing community feedback is vital for adapting campaign messages to better meet the community’s needs and concerns. Moreover, fostering community participation in campaign planning and execution can enhance the campaign’s relevance and effectiveness, making community members more likely to engage with and support the campaign’s objectives.

### 4.4 Improving vaccination service logistics

Streamlining the logistics of vaccination services can significantly enhance their accessibility and convenience, thereby improving vaccination rates. Expanding the number of vaccination sites, especially in underserved areas, and optimizing scheduling systems to reduce waiting times are key strategies. Simplifying the vaccination process through effective information systems can help individuals understand and navigate the process better, reducing any perceived complexities. Additionally, training staff to improve their interactions with the public can lead to a more positive vaccination experience, fostering a supportive atmosphere that encourages people to get vaccinated. Together, these strategies aim to create a more efficient and user-friendly vaccination infrastructure that can cater to the needs of diverse populations.

## 5 Conclusion and future work

This paper explores the influence of public health campaigns on vaccination behavior, focusing on socioeconomic status, geographical location, campaign quality, and service accessibility. The findings highlight that individuals from higher socioeconomic backgrounds are more likely to get vaccinated, emphasizing the need for targeted outreach and education to address disparities. Tailored programs for lower socioeconomic groups, aimed at removing barriers like misinformation and financial constraints, are essential for equitable vaccination uptake. Geographically, urban residents are more inclined to get vaccinated than their rural counterparts. To address this gap, mobile vaccination units, community-led initiatives, and trusted local figures can be utilized to promote vaccination in rural areas. Bringing services closer to rural populations can help overcome logistical barriers and improve vaccination rates. The research also underscores the importance of effective public health campaigns, finding that clear, culturally relevant messaging improves vaccination outcomes. Campaigns should utilize various communication channels, including social and traditional media, while collaborating with local organizations and influencers to broaden their reach. Improving the convenience of vaccination services is another crucial factor. Expanding the number of sites, reducing wait times, and simplifying the process through online appointments and transportation support can significantly increase vaccination rates. Well-equipped and staffed vaccination centers enhance the overall experience and encourage participation.

Future research should focus on misinformation and its role in vaccination hesitancy, as well as explore behavioral economics to understand how incentives influence decisions. Emerging technologies like artificial intelligence can be integrated to optimize vaccination efforts, predict behavior, and evaluate interventions. By addressing these diverse factors, vaccination rates can be increased, contributing to overall public health improvements.

Although this study provides important insights, there are still some limitations to acknowledge. First, the sample size may not be sufficient to fully represent a broader population, which could limit the generalizability of the results. Second, potential bias in survey responses may exist, such as respondents adjusting their answers due to social expectations or inaccuracies in recall. Lastly, the applicability of the findings to other regions in China may be limited, as differences in demographic characteristics, cultural backgrounds, and policy environments across regions could influence vaccination attitudes and behaviors. Therefore, future research should aim to increase the sample size and include a more diverse population to improve external validity, while also exploring the impact of regional differences on the study’s conclusions. A systematic review of global COVID-19 vaccination (22) pointed out that there are significant differences in vaccine acceptance between low-income and high-income countries, with the former often facing limitations related to healthcare system capacity and the availability of information.

## Data Availability

The original contributions presented in the study are included in the article/Supplementary material, further inquiries can be directed to the corresponding author.
