# Supplementary material for: The influence of factors related to public health campaigns on vaccination behavior among population of Wuxi region, China
Source: Front Public Health. 2025 Jan 10;12:1498296. doi: 10.3389/fpubh.2024.1498296 (PMC11757142; doi:10.3389/fpubh.2024.1498296)
Supplement: Supplementary file 1 [file Data_Sheet_1.PDF]

Questionnaire

The Influence of Public Health Campaigns on Vaccination Behaviour

**ID (For office use only)**	

Dear Participant,

We cordially invite you to participate in a research study titled "The Influence of Public Health Campaigns on Vaccination Behaviour." This questionnaire aims to gather insights into how public health campaigns affect individuals' decisions regarding vaccinations. Your participation will provide valuable data that can help improve future health communication strategies and vaccination campaigns.

Please be assured that all responses you provide will be kept strictly confidential and used solely for the purposes of this research. No personal information that could identify you will be disclosed at any point.

The questionnaire is divided into six parts, covering basic socioeconomic information, perceptions of socioeconomic status, geographical location, quality of health campaigns, vaccination convenience, and vaccination behavior. Your honest and thoughtful responses are greatly appreciated.

Thank you for considering to be part of this important study. Your input is crucial in helping us understand and enhance the effectiveness of public health initiatives.

Sincerely,

Instructions:

Please answer the following questions about yourself. Your responses will remain confidential and will be used only for research purposes.

Part 1: Basic socioeconomic information

No.	Question	Answer (Please circle or write down your answer)
1.1	When did you born? (Write down the year of birth)	
1.2	What is your gender?	1. Male<br>2. Female<br>3. Prefer not to say<br>4. Other (please specify): ___________________________
1.3	What is the highest level of education you have completed?	1. Primary school<br>2. Junior high school<br>3. Senior high school<br>4. Bachelor degree<br>5. Master degree<br>6. Doctoral degree or higher

		7. Prefer not to say
1.4	What is your marital status?	1. Single<br>2. Married<br>3. Divorced<br>4. Widowed<br>5. Prefer not to say
1.5	How many children do you have?	1. None<br>2.1<br>2.2<br>3.3<br>4. 4 and more
1.6	What is your occupation?	1. Healthcare professional (e.g., doctor, nurse, pharmacist)<br>2. Educator (e.g., teacher, professor)<br>3. Student<br>4. Government or public service employee<br>5. Private sector employee (non-healthcare)<br>6. Self-employed<br>7. Unemployed<br>8. Retired<br>9. Other (Please specify: ____________)

1.7	What is your field of work?	1. Healthcare and medical services<br>2. Education and academic research<br>3. Engineering and technology<br>4. Business and finance<br>5. Arts and entertainment<br>6. Government or public administration<br>7. Non-profit and community service<br>8. Student (if currently studying)<br>9. Unemployed/Retired (not applicable)<br>10. Other (Please specify: ____________)
1.8	What type of resident are you staying in?	1. Single-family home<br>2. Apartment/Condominium<br>3. Shared housing (e.g., dormitory, roommate situation)<br>4. Assisted living facility (e.g., retirement home)<br>5. Temporary housing (e.g., hotel, hostel)<br>6. Homeless or without stable housing

Part 2: Socioeconomic perception

No.	Statement	Response (Please circle the best response fitting to your opinion)
2.1	My employment status/occupation provides me with adequate resources and flexibility for healthcare decisions, including vaccination.	1. Strongly disagree<br>2. Disagree<br>3. Neither agree nor disagree<br>4. Agree<br>5. Strongly Agree
2.2	Due to my socioeconomic status, I have easy access to reliable health information and resources, including vaccination.	1. Strongly disagree<br>2. Disagree<br>3. Neither agree nor disagree<br>4. Agree<br>5. Strongly Agree
2.3	I am confident that I can continue my current job until I retire.	1. Strongly disagree<br>2. Disagree<br>3. Neither agree nor disagree<br>4. Agree

		5. Strongly Agree
2.4	I am confident that I can keep my financial status stable for as long as I wish.	1. Strongly disagree<br>2. Disagree<br>3. Neither agree nor disagree<br>4. Agree<br>5. Strongly Agree
2.5	The neighbourhood I live in is clean and conducive in promoting physical, mental, and social health.	1. Strongly disagree<br>2. Disagree<br>3. Neither agree nor disagree<br>4. Agree<br>5. Strongly Agree
2.6	The neighbourhood I live in has a very strong social support network, especially in matters related to health.	1. Strongly disagree<br>2. Disagree<br>3. Neither agree nor disagree<br>4. Agree<br>5. Strongly Agree
2.7	What is your employment status?	1. Permanent<br>2. Contract<br>3. Trainee<br>4. Self-employed<br>5. Other (please specify):<br>______________________________
2.8	What is your annual income?	1. Under ¥20,000

		2. ¥20,000 to ¥39,999<br><br>3. ¥40,000 to ¥59,999<br><br>4. ¥60,000 to ¥79,999<br><br>5. Over ¥80,000

Part 3: Geographical location perception

No.	Statement	Response<br>(Please circle the best response fitting to your opinion)
3.1	I am living in an urban community.	1. Strongly disagree<br>2. Disagree<br>3. Neither agree nor disagree<br>4. Agree<br>5. Strongly Agree
3.2	Living in the current area provides me with close proximity to healthcare services, including vaccination centres.	1. Strongly disagree<br>2. Disagree<br>3. Neither agree nor disagree<br>4. Agree<br>5. Strongly Agree
3.3	In my current community, there is a prevalent positive attitude towards vaccination, which also influences my decision to take vaccination.	1. Strongly disagree<br>2. Disagree<br>3. Neither agree nor disagree<br>4. Agree<br>5. Strongly Agree
3.4	The community lifestyle in my living area facilitates easier management and scheduling	1. Strongly disagree<br>2. Disagree

	of health-related activities, including vaccination.	3. Neither agree nor disagree<br>4. Agree<br>5. Strongly Agree
3.5	In the past six months, how many times have you been exposed to public health campaigns that promote vaccination?	1. None<br>2.1<br>2.2<br>3.3<br>4. 4 and more

Part 4: Quality of health campaign perception

Note: For this Part, please recall the last public health campaign related to vaccination in your area that you have encountered and give your opinion on the following statements.

No.	Statement	Response<br>(Please circle the best response fitting to your opinion.)
4.1	The information provided by the public health campaign about vaccination was accurate and reliable.	1. Strongly disagree<br>2. Disagree<br>3. Neither agree nor disagree<br>4. Agree<br>5. Strongly Agree
4.2	The public health campaign based their vaccination information on solid scientific evidence.	1. Strongly disagree<br>2. Disagree<br>3. Neither agree nor disagree<br>4. Agree<br>5. Strongly Agree
4.3	The vaccination information provided by the public health campaign was easy to understand.	1. Strongly disagree<br>2. Disagree<br>3. Neither agree nor disagree

		4. Agree<br>5. Strongly Agree
4.4	The vaccination information provided by the public health campaign was clear.	1. Strongly disagree<br>2. Disagree<br>3. Neither agree nor disagree<br>4. Agree<br>5. Strongly Agree
4.5	The quality of the public health campaign has positively influenced my attitude towards vaccination.	1. Strongly disagree<br>2. Disagree<br>3. Neither agree nor disagree<br>4. Agree<br>5. Strongly Agree
4.6	The information in the public health campaign was in line with our social norms.	1. Strongly disagree<br>2. Disagree<br>3. Neither agree nor disagree<br>4. Agree<br>5. Strongly Agree

Part 5: Vaccination convenience perception

No.	Statement	Response<br>(Please circle the best response fitting to your opinion.)
5.1	In my area, vaccination sites are easily accessible and conveniently located.	1. Strongly disagree<br>2. Disagree<br>3. Neither agree nor disagree<br>4. Agree<br>5. Strongly Agree
5.2	I usually experience minimal waiting times when I go for vaccination.	1. Strongly disagree<br>2. Disagree<br>3. Neither agree nor disagree<br>4. Agree<br>5. Strongly Agree
5.3	The process and procedures for getting vaccinated in my area are straightforward and easy.	1. Strongly disagree<br>2. Disagree<br>3. Neither agree nor disagree

		4. Agree<br>5. Strongly Agree
5.4	I find it easy to access clear and helpful information about vaccination schedules and procedures.	1. Strongly disagree<br>2. Disagree<br>3. Neither agree nor disagree<br>4. Agree<br>5. Strongly Agree
5.5	The healthcare staff in the vaccination sites are very approachable and professional.	1. Strongly disagree<br>2. Disagree<br>3. Neither agree nor disagree<br>4. Agree<br>5. Strongly Agree

Part 6: Vaccination behaviour

No.	Statement	Response<br>(Please circle the best response fitting to your opinion.)
6.1	I am willing to receive vaccinations that are recommended by official public health campaigns.	1. Strongly disagree<br>2. Disagree<br>3. Neither agree nor disagree<br>4. Agree<br>5. Strongly Agree
6.2	How many times have you been vaccinated after 18 years old?	1. None<br>2.1<br>2.2<br>3.3<br>4. 4 and more
6.3	I plan to adhere to future vaccination recommendations by public health campaigns, including booster shots and new vaccines.	1. Strongly disagree<br>2. Disagree<br>3. Neither agree nor disagree<br>4. Agree<br>5. Strongly Agree
